# Supplementary material for: Understanding no-show behaviour for cervical cancer screening appointments among hard-to-reach women in Bogotá, Colombia: A mixed-methods approach
Source: PLoS One. 2022 Jul 22;17(7):e0271874. doi: 10.1371/journal.pone.0271874 (PMC9307170; doi:10.1371/journal.pone.0271874)
Supplement: S1 Appendix — (DOCX) [file pone.0271874.s001.docx]

**S1 Appendix. Interview guide**

Question 1: Could you describe your experience using health services, over the last three years?

Question 2: On (insert date) a community worker visited your home. Could you describe the visit?

Question 3: Do you think is important having a cervical cytology? Why?

Question 4: According to our records, you did not attend the cytology appointment that was scheduled on (insert date). Could you tell me why?

Question 5: In your opinion, which other reasons could prevent a patient to keep her cytology appointment?

Question 6: Have any of your close friends or relatives had a cytology? If so, what have they told you about this experience?

Question 7: During the home visit, did the community worker discussed with you the importance of having a cytology? Do you remember what did she/he tell you?

Question 8: What do think it can be done to increase cytology uptake?

Question 9: Is there anything else you want to tell me about the experience of having a cervical cytology?
