# Supplementary material for: Understanding no-show behaviour for cervical cancer screening appointments among hard-to-reach women in Bogotá, Colombia: A mixed-methods approach
Source: PLoS One. 2022 Jul 22;17(7):e0271874. doi: 10.1371/journal.pone.0271874 (PMC9307170; doi:10.1371/journal.pone.0271874)
Supplement: S2 Appendix — (DOCX) [file pone.0271874.s002.docx]

**S2 Appendix. Analytical Framework Categories**

**Table1: First order categories description**

| Second order | First order | | Description |
| --- | --- | --- | --- |
| Barriers | Access | |  |
|  | 1 | Financial stress | Economic constrains such as out-out-pocket payments. |
|  | 2 | Inconvenient appointment slots | Patient finds it difficult to attend an appointment on the available slots. |
|  | 3 | Long lead times | The time elapsed between the appointment scheduling and the actual appointment date is too long. |
|  | 4 | Geographical access | Patients finds it difficult to reach the appointment facility location. |
|  | 5 | Work Commitments | Work obligations make it difficult to attend medical appointments. |
|  | Service delivery | |  |
|  | 6 | Bad experiences with service delivery | Prior negative experiences while using healthcare services. |
|  | 7 | Bad experiences with home visit | Negative experiences during ACS home visits. |
|  | 8 | Communication | Confusing or absent information about appointments or exams. |
|  | 9 | Dismissive staff | Rude or disrespectful behaviours from health care providers. |
|  | 10 | Lack of flexibility in service delivery | Incapability to adapt or modify the service delivery process according to the patient’s needs. |
|  | 11 | Lack of information during the home visit | Incomplete or confusing information, during home, about the exam or the service delivery. |
|  | 12 | Multiple appointments | Patient has multiple appointments to attend at the same day. |
|  | 13 | Poor care quality | Patients perceive a low quality in the health services. |
|  | 14 | Prefers to use other care | Patients attend to other medical service or types of healthcare approaches. |
|  | 15 | Process design | Challenges related to the steps or requirements to book or attend an appointment. |
|  | Personal | |  |
|  | 16 | Family care | Demands on women's time including child-care and housework. |
|  | 17 | Forgetfulness | Patients forget the appointment. |
|  | 18 | Health issues | The patient is experiencing health problems and decided not to attend. |
|  | 19 | Lack of network support | Women do not have the support of their partner or family to carry out the exam. |
|  | 20 | Language | Inability to communicate effectively due to lack of fluency in the language. |
|  | 21 | Migration | Patients move to another city or country. |
|  | 22 | Other priorities | Women decided to prioritize another task over the appointment. |
|  | 23 | Religion | Religious beliefs prevent them from attending the exam. |
|  | 24 | Travel | Patient was traveling at the appointment’s date. |
| Second order | First order | | Description |
| Barriers | Protective behaviour | |  |
|  | 25 | Anxiety | Feelings of anxiety towards the procedure. |
|  | 26 | Non-compliance with requirements | Failure to comply with the requirements for screening (i.e., having had sexual intercourses on the last 24 hours). |
|  | 27 | Discomfort | Perception that the screening procedure is uncomfortable. |
|  | 28 | Embarrassment | Feelings of embarrassment about the cervical examination. |
|  | 29 | Gender of the health provider | Women's preferences regarding the gender of the health care provider. |
|  | 30 | Pain | Perception that the screening procedure is painful. |
|  | 31 | Peer influence | Experiences of friends or peers influences the preferences for screening. |
| Benefits | Protective Behaviour | |  |
|  | 32 | Cancer diagnosis | Recognition of the possibility of diagnosing cancer. |
|  | 33 | Health | Belief that screening is beneficial for health. |
|  | 34 | Lack of perceived benefits | Patient does not perceive benefits on screening participation. |
|  | 35 | Lack of knowledge | Patient does not know what screening is, why is important or have received misleading information. |
|  | 36 | Screening program | Patient does not have information about the screening program. |
|  | Service delivery | |  |
|  | 37 | Satisfaction (home visit) | Patient satisfaction with the home visit. |
|  | 38 | Satisfaction (service delivery) | Patient satisfaction with the healthcare service delivery. |
| Susceptibility | 39 | Perceived susceptibility | Patient perception of her own risk of developing cervical cancer. |
|  | 40 | Denial | Patients deny they might need medical attention. |
| Severity | 41 | Fear of a bad result | Fear of the outcome of the test. |
|  | 42 | Fear of side effects | Fear of experiencing undesirable and unintended effects of the test. |
|  | 43 | Only uses emergency care | Lack of familiarity with preventative health and tendency to seek health services only when ill. |
|  | 44 | Severity of the consequences | Patient perceives that the consequences of developing cervical cancer are severe. |

**Table2: References supporting the first order categories**

| Second order | First order | | | No-Show behaviour studies | Cervical cancer screening studies |  |
| --- | --- | --- | --- | --- | --- | --- |
| Barriers | Access | | |  |  |  |
|  | 1 | Financial stress | | [1], [2], [11], [12], [3]–[10] | [13], [14], [23], [15]–[22] | |
|  | 2 | Inconvenient appointment slots | | Inductive category* | Inductive category* | |
|  | 3 | Long lead times | | [2], [4], [29]–[38], [7], [39]–[42], [9], [10], [24]–[28] | [13], [14], [45]–[52], [18]–[23], [43], [44] | |
|  | 4 | Geographical access | | [1], [2], [25], [27]–[29], [31], [34], [35], [37], [39], [53], [3], [54]–[63], [4], [64]–[66], [5], [7], [8], [10], [12], [24] | [13], [15], [68], [19], [23], [44], [45], [47]–[49], [67] | |
|  | 5 | Work Commitments | | [1], [3], [55], [57], [59], [61], [63], [66], [69], [70], [12], [31], [32], [36], [40], [41], [53], [54] | [21], [22], [44] | |
|  | Service delivery | | |  |  | |
|  | 6 | Bad experiences with service delivery | | [2], [3], [25], [62], [71], [72] | [16], [17], [19]–[22], [45], [73], [74] | |
|  | 7 | Bad experiences with home visit | | Inductive category* | Inductive category* | |
|  | 8 | Communication | | [4], [5], [56], [61], [63]–[65], [69], [75]–[77], [8], [25], [27], [30], [35], [36], [38], [40] | [16], [19], [21], [45] | |
|  | 9 | Dismissive staff | | [2], [11], [25], [54], [65], [72] | - | |
|  | 10 | Lack of flexibility in service delivery | | Inductive category* | Inductive category* | |
|  | 11 | Lack of information during the home visit | | Inductive category* | Inductive category* | |
|  | 12 | Multiple appointments | | [31], [42] | - | |
|  | 13 | Poor care quality | | [32], [54] | - | |
| - | 14 | Prefers to use other care | | [54], [58] | [15], [78] | |
|  | 15 | Process design | | [28], [31], [64] | [15], [21], [22], [45], [48] | |
|  | Personal | | |  |  | |
|  | 16 | Family care | | [1], [2], [55], [58], [60], [63], [65], [66], [11], [12], [25], [31], [35], [36], [41], [54] | [51], [67], [79] | |
|  | 17 | Forgetfulness | | [2], [8], [58], [59], [61], [65], [66], [77], [12], [25], [31], [32], [34], [40], [56], [57] | - | |
|  | 18 | Health issues | | [3], [8], [66], [70], [75], [11], [12], [25], [31], [39], [41], [55], [60] | - | |
|  | 19 | Lack of network support | | [2], [7], [8], [24], [28], [60], [69], [80] | [15], [18], [21], [49], [68], [81], [82] | |
|  | 20 | Language | | [1] | [48], [78], [83], [84] | |
|  | 21 | Migration | | [1] | - | |
|  | 22 | Other priorities | | [3], [25], [38], [39], [57], [60], [61] | - | |
|  | 23 | Religion | | [24], [29], [54], [85] | [67], [84] | |
|  | 24 | Travel | | [24], [41], [65] | - | |
| Second order | First order | | No-Show behaviour studies | | Cervical cancer screening studies | |
| Barriers | Protective behaviour | |  | |  | |
|  | 25 | Anxiety | [34], [39], [65], [70], [86] | | - | |
|  | 26 | Non-compliance with requirements | Inductive category* | | Inductive category* | |
|  | 27 | Discomfort | [56] | | - | |
|  | 28 | Embarrassment | - | | [14], [17], [78], [79], [87], [22], [44], [51], [52], [67], [68], [73], [74] | |
|  | 29 | Gender of the health provider | - | | [14], [15], [52], [79], [81], [82], [17], [20], [21], [45], [47]–[49], [51] | |
|  | 30 | Pain | - | | [14], [17], [87], [88], [44], [46], [47], [51], [52], [73], [79], [81] | |
|  | 31 | Peer influence | [2], [7], [54], [56] | | [67], [68] | |
| Benefits | Protective Behaviour | |  | |  | |
|  | 32 | Cancer diagnosis | Inductive category* | | Inductive category* | |
|  | 33 | Health | Inductive category* | | Inductive category* | |
|  | 34 | Lack of perceived benefits | [3], [6], [71], [72], [77], [7], [9], [33], [35], [53], [54], [57], [60] | | [13], [18], [89], [44], [46], [47], [73], [74], [79], [87], [88] | |
|  | 35 | Lack of knowledge | - | | [13], [14], [48], [49], [51], [52], [67], [68], [73], [74], [78], [82], [16], [84], [87]–[91], [17]–[19], [22], [43], [44], [46] | |
|  | 36 | Screening program | Inductive category* | | Inductive category* | |
|  | Service delivery | |  | |  | |
|  | 37 | Satisfaction (home visit) | Inductive category* | | Inductive category* | |
|  | 38 | Satisfaction (service delivery) | Inductive category* | | Inductive category* | |
| Susceptibility | 39 | Perceived susceptibility | - | | [16], [18], [81], [87], [89], [91] | |
|  | 40 | Denial | [26], [75] | | - | |
| Severity | 41 | Fear of a bad result | [4], [34]–[37], [71], [72], [75] | | [14], [15], [78], [79], [82], [17], [21], [22], [47], [48], [52], [67], [74] | |
|  | 42 | Fear of side effects | - | | [17], [82], [88] | |
|  | 43 | Only uses emergency care | [54], [71] | | - | |
|  | 44 | Severity of the consequences | [24], [33], [53], [63] | | [14], [17], [68], [79], [81], [84], [90], [91], [19], [21], [22], [43], [44], [48], [52], [67] | |

* A first order category is inductive if it emerged from the data and was not identified in the literature reviews.

**References**

[1] E. R. Wolf *et al.*, “Caregiver and clinician perspectives on missed well-child visits,” *Ann. Fam. Med.*, vol. 18, no. 1, pp. 30–34, 2020, doi: 10.1370/afm.2466.

[2] M. I. Heaman *et al.*, “Barriers and facilitators related to use of prenatal care by inner-city women: Perceptions of health care providers,” *BMC Pregnancy Childbirth*, vol. 15, no. 1, pp. 1–13, 2015, doi: 10.1186/s12884-015-0431-5.

[3] E. Cameron *et al.*, “Health care professionals’ views of paediatric outpatient non-attendance: Implications for general practice,” *Fam. Pract.*, vol. 31, no. 1, pp. 111–117, 2014, doi: 10.1093/fampra/cmt063.

[4] C. R. Freed, S. T. Hansberry, and M. I. Arrieta, “Structural and hidden barriers to a local primary health care infrastructure: Autonomy, decisions about primary health care, and the centrality and significance of power,” *Res. Sociol. Health Care*, vol. 31, no. 2013, pp. 57–81, 2013, doi: 10.1108/S0275-4959(2013)0000031006.

[5] M. D. Morris, S. T. Popper, T. C. Rodwell, S. K. Brodine, and K. C. Brouwer, “Healthcare barriers of refugees post-resettlement,” *J. Community Health*, vol. 34, no. 6, pp. 529–538, 2009, doi: 10.1007/s10900-009-9175-3.

[6] E. Pegon-Machat, S. Tubert-Jeannin, C. Loignon, A. Landry, and C. Bedos, “Dentists’ experience with low-income patients benefiting from a public insurance program,” *Eur. J. Oral Sci.*, vol. 117, no. 4, pp. 398–406, 2009, doi: 10.1111/j.1600-0722.2009.00643.x.

[7] A. Topuzoǧlu, P. Ay, S. Hidiroglu, and Y. Gurbuz, “The barriers against childhood immunizations: A qualitative research among socio-economically disadvantaged mothers,” *Eur. J. Public Health*, vol. 17, no. 4, pp. 348–352, 2007, doi: 10.1093/eurpub/ckl250.

[8] K. D. Gashu, K. A. Gelaye, and B. Tilahun, “Adherence to TB treatment remains low during continuation phase among adult patients in Northwest Ethiopia,” *BMC Infect. Dis.*, vol. 21, no. 1, pp. 1–10, 2021, doi: 10.1186/s12879-021-06428-6.

[9] S. Leijdesdorff, R. Klaassen, D.-J. Wairata, S. Rosema, T. van Amelsvoort, and A. Popma, “Barriers and facilitators on the pathway to mental health care among 12-25 year olds,” *Int. J. Qual. Stud. Health Well-being*, vol. 16, no. 1, p. 1963110, 2021, doi: 10.1080/17482631.2021.1963110.

[10] J. E. Sherbuk *et al.*, “A qualitative study of perceived barriers to hepatitis C care among people who did not attend appointments in the non-urban US South,” *Harm Reduct. J.*, vol. 17, no. 1, p. 64, 2020, doi: 10.1186/s12954-020-00409-9.

[11] M. Yang *et al.*, “Qualitative Analyses of the Reasons Why Patients Do Not Attend Scheduled Inpatient Appointments in a Hospital in Guangzhou, China.,” *Risk Manag. Healthc. Policy*, vol. 13, pp. 2857–2865, 2020, doi: 10.2147/RMHP.S280665.

[12] S. Ofei-Dodoo, R. Kellerman, C. Hartpence, K. Mills, and E. Manlove, “Why Patients Miss Scheduled Outpatient Appointments at Urban Academic Residency Clinics,” *Kansas J. Med.*, vol. 12, no. 3, pp. 57–61, 2019, doi: 10.17161/kjm.v12i3.11793.

[13] H. Lee *et al.*, “Exploring Complicity of Cervical Cancer Screening in Malawi: The Interplay of Behavioral, Cultural, and Societal Influences,” *Asia-Pacific J. Oncol. Nurs.*, vol. 7, no. 1, pp. 18–27, Dec. 2019, doi: 10.4103/apjon.apjon_48_19.

[14] C. Binka, S. H. Nyarko, K. Awusabo-Asare, and D. T. Doku, “Barriers to the Uptake of Cervical Cancer Screening and Treatment among Rural Women in Ghana,” *Biomed Res. Int.*, vol. 2019, p. 6320938, 2019, doi: 10.1155/2019/6320938.

[15] A. O. C. Onyenwenyi and G. G. McHunu, “Barriers to cervical cancer screening uptake among rural women in South West Nigeria: A qualitative study,” *S. Afr. J. Obstet. Gynaecol.*, vol. 24, no. 1, pp. 22–26, 2018, doi: 10.7196/SAJOG.2018.v24i1.1290.

[16] N. E. Schoenberg, T. M. Kruger, S. Bardach, and B. M. Howell, “Appalachian women’s perspectives on breast and cervical cancer screening.,” *Rural Remote Health*, vol. 13, no. 3, p. 2452, 2013.

[17] O. T. Hasahya, V. Berggren, D. Sematimba, R. C. Nabirye, and E. Kumakech, “Beliefs, perceptions and health-seeking behaviours in relation to cervical cancer: A qualitative study among women in Uganda following completion of an HPV vaccination campaign,” *Glob. Health Action*, vol. 9, no. 1, pp. 1–9, 2016, doi: 10.3402/gha.v9.29336.

[18] A. Adedimeji *et al.*, “Challenges and opportunities associated with cervical cancer screening programs in a low income, high HIV prevalence context,” *BMC Womens. Health*, vol. 21, no. 1, pp. 1–14, 2021, doi: 10.1186/s12905-021-01211-w.

[19] A. N. Roux *et al.*, “Barriers to cervical cancer prevention in rural Cameroon: A qualitative study on healthcare providers’ perspective,” *BMJ Open*, vol. 11, no. 6, pp. 1–8, 2021, doi: 10.1136/bmjopen-2020-043637.

[20] S. S. Mkhonta and J. Shirinde, “Registered nurses’ perspectives on barriers of cervical cancer screening in swaziland: A qualitative study,” *Pan Afr. Med. J.*, vol. 38, pp. 1–12, 2021, doi: 10.11604/pamj.2021.38.295.22431.

[21] L. Vasudevan *et al.*, “Barriers to the uptake of cervical cancer services and attitudes towards adopting new interventions in Peru,” *Prev. Med. Reports*, vol. 20, p. 101212, 2020, doi: 10.1016/j.pmedr.2020.101212.

[22] F. Christie-de Jong and S. Reilly, “Barriers and facilitators to pap-testing among female overseas Filipino workers: a qualitative exploration,” *Int. J. Hum. Rights Healthc.*, vol. 13, no. 3, pp. 275–288, 2020, doi: 10.1108/IJHRH-01-2020-0006.

[23] J. L. Moss *et al.*, “Multilevel Associations with Cancer Screening Among Women in Rural, Segregated Communities Within the Northeastern USA: a Mixed-Methods Study,” *J. Cancer Educ.*, no. 0123456789, 2021, doi: 10.1007/s13187-021-02069-0.

[24] M. M. Gombe *et al.*, “Key barriers and enablers associated with uptake and continuation of oral pre-exposure prophylaxis (PrEP) in the public sector in Zimbabwe: Qualitative perspectives of general population clients at high risk for HIV,” *PLoS One*, vol. 15, no. 1, pp. 1–18, 2020, doi: 10.1371/journal.pone.0227632.

[25] M. Ballantyne, L. Liscumb, E. Brandon, J. Jaffar, A. Macdonald, and L. Beaune, “Mothers’ Perceived Barriers to and Recommendations for Health Care Appointment Keeping for Children Who Have Cerebral Palsy,” *Glob. Qual. Nurs. Res.*, vol. 6, 2019, doi: 10.1177/2333393619868979.

[26] P. Gellasch, “The Developmental Screening Behaviors, Skills, Facilitators, and Constraints of Family Nurse Practitioners in Primary Care: A Qualitative Descriptive Study,” *J. Pediatr. Heal. Care*, vol. 33, no. 4, pp. 466–477, Jul. 2019, doi: 10.1016/j.pedhc.2019.01.004.

[27] M. Dilgul, P. McNamee, S. Orfanos, C. E. Carr, and S. Priebe, “Why do psychiatric patients attend or not attend treatment groups in the community: A qualitative study,” *PLoS One*, vol. 13, no. 12, pp. 1–16, 2018, doi: 10.1371/journal.pone.0208448.

[28] S. G. Minick *et al.*, “Participants’ perspectives on improving retention in HIV care after hospitalization: A post-study qualitative investigation of the MAPPS study,” *PLoS One*, vol. 13, no. 8, pp. 1–14, 2018, doi: 10.1371/journal.pone.0202917.

[29] F. L. Cavallaro *et al.*, “Understanding ‘missed appointments’ for pills and injectables: A mixed methods study in Senegal,” *BMJ Glob. Heal.*, vol. 3, no. 6, 2018, doi: 10.1136/bmjgh-2018-000975.

[30] D. Marshall *et al.*, “What IAPT services can learn from those who do not attend,” *J. Ment. Heal.*, vol. 25, no. 5, pp. 410–415, 2016, doi: 10.3109/09638237.2015.1101057.

[31] J. Touch and J. P. Berg, “Parent perspectives on appointment nonattendance: A descriptive study,” *Pediatr. Nurs.*, vol. 42, no. 4, pp. 181–188, 2016.

[32] Y. Lam *et al.*, “Provider-level and other health systems factors influencing engagement in HIV care: A qualitative study of a vulnerable population,” *PLoS One*, vol. 11, no. 7, pp. 1–14, 2016, doi: 10.1371/journal.pone.0158759.

[33] N. Schwennesen, J. E. Henriksen, and I. Willaing, “Patient explanations for non-attendance at type 2 diabetes self-management education: A qualitative study,” *Scand. J. Caring Sci.*, vol. 30, no. 1, pp. 187–192, 2016, doi: 10.1111/scs.12245.

[34] R. Strutton, A. Du Chemin, I. M. Stratton, and A. S. Forster, “System-level and patient-level explanations for non-attendance at diabetic retinopathy screening in Sutton and Merton (London, UK): A qualitative analysis of a service evaluation,” *BMJ Open*, vol. 6, no. 5, pp. 1–6, 2016, doi: 10.1136/bmjopen-2015-010952.

[35] V. B. Sheppard, J. Huei-yu Wang, J. Eng-Wong, S. H. Martin, A. Hurtado-de-Mendoza, and G. Luta, “Promoting mammography adherence in underserved women: the telephone coaching adherence study.,” *Contemp. Clin. Trials*, vol. 35, no. 1, pp. 35–42, May 2013, doi: 10.1016/j.cct.2013.02.005.

[36] A. Sinclair and H. A. Alexander, “Using outreach to involve the hard-to-reach in a health check: What difference does it make?,” *Public Health*, vol. 126, no. 2, pp. 87–95, 2012, doi: 10.1016/j.puhe.2011.11.004.

[37] L. M. Bollinger, K. G. Nire, M. M. Rhodes, D. J. Chisolm, and S. H. O’Brien, “Caregivers’ perspectives on barriers to transcranial Doppler screening in children with sickle-cell disease.,” *Pediatr. Blood Cancer*, vol. 56, no. 1, pp. 99–102, Jan. 2011, doi: 10.1002/pbc.22780.

[38] C. Martin, T. Perfect, and G. Mantle, “Non-attendance in primary care: The views of patients and practices on its causes, impact and solutions,” *Fam. Pract.*, vol. 22, no. 6, pp. 638–643, 2005, doi: 10.1093/fampra/cmi076.

[39] T. D. Denberg *et al.*, “Predictors of nonadherence to screening colonoscopy,” *J. Gen. Intern. Med.*, vol. 20, no. 11, pp. 989–995, 2005, doi: 10.1111/j.1525-1497.2005.00164.x.

[40] H. Alderson *et al.*, “Using behavioural insights to improve the uptake of services for drug and alcohol misuse,” *Int. J. Environ. Res. Public Health*, vol. 18, no. 13, 2021, doi: 10.3390/ijerph18136923.

[41] S. Chamberlin, M. Mphande, K. Phiri, P. Kalande, and K. Dovel, “How HIV Clients Find Their Way Back to the ART Clinic: A Qualitative Study of Disengagement and Re-engagement with HIV Care in Malawi,” *AIDS Behav.*, no. 0123456789, 2021, doi: 10.1007/s10461-021-03427-1.

[42] C.-A. Christie-Johnston, R. O’Loughlin, and H. Hiscock, “‘Getting to clinic study’: A mixed methods study of families who fail to attend hospital outpatient clinics,” *J. Paediatr. Child Health*, vol. 56, no. 4, pp. 506–511, Apr. 2020, doi: https://doi.org/10.1111/jpc.14672.

[43] T. Brandt *et al.*, “Genital self-sampling for HPV-based cervical cancer screening: a qualitative study of preferences and barriers in rural Ethiopia,” *BMC Public Health*, vol. 19, no. 1, p. 1026, 2019, doi: 10.1186/s12889-019-7354-4.

[44] T. G. Matenge and B. Mash, “Barriers to accessing cervical cancer screening among HIV positive women in Kgatleng district, Botswana: A qualitative study,” *PLoS One*, vol. 13, no. 10, pp. 1–13, 2018, doi: 10.1371/journal.pone.0205425.

[45] C. Gu *et al.*, “Understanding the cervical screening behaviour of Chinese women: The role of health care system and health professions,” *Appl. Nurs. Res.*, vol. 39, no. September 2016, pp. 58–64, 2018, doi: 10.1016/j.apnr.2017.09.009.

[46] C. Curmi, K. Peters, and Y. Salamonson, “Barriers to cervical cancer screening experienced by lesbian women: a qualitative study,” *J. Clin. Nurs.*, vol. 25, no. 23–24, pp. 3643–3651, 2016, doi: 10.1111/jocn.12947.

[47] C. Malhotra, M. Bilger, J. Liu, and E. Finkelstein, “Barriers to Breast and Cervical Cancer Screening in Singapore: a Mixed Methods Analysis.,” *Asian Pac. J. Cancer Prev.*, vol. 17, no. 8, pp. 3887–3895, 2016.

[48] M. Vahabi and A. Lofters, “Muslim immigrant women’s views on cervical cancer screening and HPV self-sampling in Ontario, Canada,” *BMC Public Health*, vol. 16, no. 1, pp. 1–13, 2016, doi: 10.1186/s12889-016-3564-1.

[49] A. C. Munthali, B. M. Ngwira, and F. Taulo, “Exploring barriers to the delivery of cervical cancer screening and early treatment services in Malawi: Some views from service providers,” *Patient Prefer. Adherence*, vol. 9, pp. 501–508, 2015, doi: 10.2147/PPA.S69286.

[50] S. A. Francis, K. A. Leser, E. E. Esmont, and F. M. Griffith, “An analysis of key stakeholders’ attitudes and beliefs about barriers and facilitating factors in the development of a cervical cancer prevention program in South Africa.,” *Afr. J. Reprod. Health*, vol. 17, no. 1, pp. 158–168, 2013.

[51] L. Logan and S. McIlfatrick, “Exploring women’s knowledge, experiences and perceptions of cervical cancer screening in an area of social deprivation,” *Eur. J. Cancer Care (Engl).*, vol. 20, no. 6, pp. 720–727, 2011, doi: 10.1111/j.1365-2354.2011.01254.x.

[52] K. Adewumi, H. Nishimura, S. Y. Oketch, P. Adsul, and M. Huchko, “Barriers and Facilitators to Cervical Cancer Screening in Western Kenya: a Qualitative Study,” *J. Cancer Educ.*, 2021, doi: 10.1007/s13187-020-01928-6.

[53] C. Eades and H. Alexander, “A mixed-methods exploration of non-attendance at diabetes appointments using peer researchers,” *Heal. Expect.*, vol. 22, no. 6, pp. 1260–1271, 2019, doi: 10.1111/hex.12959.

[54] W. Alanazy, J. Rance, and A. Brown, “Exploring maternal and health professional beliefs about the factors that affect whether women in Saudi Arabia attend antenatal care clinic appointments,” *Midwifery*, vol. 76, pp. 36–44, 2019, doi: 10.1016/j.midw.2019.05.012.

[55] I. S. Klatte, S. Harding, and S. Roulstone, “Speech and language therapists’ views on parents’ engagement in Parent–Child Interaction Therapy (PCIT),” *Int. J. Lang. Commun. Disord.*, vol. 54, no. 4, pp. 553–564, 2019, doi: 10.1111/1460-6984.12459.

[56] A. Fägerstad, J. Lundgren, K. Arnrup, and E. Carlson, “Barriers and facilitators for adolescent girls to take on adult responsibility for dental care–a qualitative study,” *Int. J. Qual. Stud. Health Well-being*, vol. 14, no. 1, 2019, doi: 10.1080/17482631.2019.1678971.

[57] S. Copeland, J. Muir, and A. Turner, “Understanding Indigenous patient attendance: A qualitative study,” *Aust. J. Rural Health*, vol. 25, no. 5, pp. 268–274, 2017, doi: 10.1111/ajr.12348.

[58] B. P. Magadzire, T. Mathole, and K. Ward, “Reasons for missed appointments linked to a public-sector intervention targeting patients with stable chronic conditions in South Africa: Results from in-depth interviews and a retrospective review of medical records,” *BMC Fam. Pract.*, vol. 18, no. 1, pp. 1–10, 2017, doi: 10.1186/s12875-017-0655-8.

[59] L. R. French, K. M. Turner, D. J. Sharp, H. Morley, L. Goldsworthy, and J. Hamilton-Shield, “Characteristics of children who do not attend their hospital appointments, and GPs’ response: a mixed methods study in primary and secondary care,” *Br. J. Gen. Pract.*, vol. 67, no. 660, pp. e483–e489, 2017, doi: 10.3399/bjgp17x691373.

[60] R. Poll, P. Allmark, and A. M. Tod, “Reasons for missed appointments with a hepatitis C outreach clinic: A qualitative study,” *Int. J. Drug Policy*, vol. 39, pp. 130–137, 2017, doi: 10.1016/j.drugpo.2015.12.027.

[61] N. J. Cibulka, H. W. Fischer, and A. J. Fischer, “Improving communication with low-income women using today’s technology.,” *Online J. Issues Nurs.*, vol. 17, no. 2, p. 9, Mar. 2012.

[62] N. L. Lacy, A. Paulman, M. D. Reuter, and B. Lovejoy, “Why we don’t come: Patient perceptions on no-shows,” *Ann. Fam. Med.*, vol. 2, no. 6, pp. 541–545, 2004, doi: 10.1370/afm.123.

[63] M. Saleh, J. Caron, S. Hernandez, and L. Boyd, “Determinants of Clinic Absenteeism in Gynecologic Oncology Clinic at a Safety Net Hospital,” *J. Community Health*, vol. 46, no. 2, pp. 399–404, 2021, doi: 10.1007/s10900-020-00942-5.

[64] L. Jefferson *et al.*, “Non-attendance at urgent referral appointments for suspected cancer: a qualitative study to gain understanding from patients and GPs,” *Br. J. Gen. Pract.*, vol. 69, no. 689, p. e850 LP-e859, Dec. 2019, doi: 10.3399/bjgp19X706625.

[65] C. DuMontier, K. Rindfleisch, J. Pruszynski, and J. J. 3rd Frey, “A multi-method intervention to reduce no-shows in an urban residency clinic.,” *Fam. Med.*, vol. 45, no. 9, pp. 634–641, Oct. 2013.

[66] W. N. Feitsma, R. Popping, and D. E. M. C. Jansen, “No-show at a forensic psychiatric outpatient clinic: Risk factors and reasons,” *Int. J. Offender Ther. Comp. Criminol.*, vol. 56, no. 1, pp. 96–112, 2012, doi: 10.1177/0306624X10389435.

[67] V. H. Rasul, M. A. Cheraghi, and Z. B. Moghdam, “Barriers to cervical cancer screening among Iraqi Kurdish women: A qualitative study,” *Acta Medica Mediterr.*, vol. 32, no. SpecialIssue4, pp. 1249–1256, 2016.

[68] J. Greibe Andersen, A. D. Shrestha, B. Gyawali, D. Neupane, and P. Kallestrup, “Barriers and facilitators to cervical cancer screening uptake among women in Nepal–a qualitative study,” *Women Heal.*, vol. 60, no. 9, pp. 963–974, 2020, doi: 10.1080/03630242.2020.1781742.

[69] I. Zanardelli and N. Robinson, “Factors that influence patients’ decisions to discontinue with an acupuncture service—A qualitative study,” *Eur. J. Integr. Med.*, vol. 25, no. August 2018, pp. 92–99, 2019, doi: 10.1016/j.eujim.2018.12.004.

[70] T. Britton and N. Robinson, “Pitfalls and Pearls of Wisdom in 18F-FDG PET Imaging of Tumors.,” *J. Nucl. Med. Technol.*, vol. 44, no. 2, pp. 59–64, Jun. 2016, doi: 10.2967/jnmt.115.170803.

[71] M. Dahl, J. Lindholt, R. Søgaard, L. Frost, L. S. Andersen, and V. Lorentzen, “An interview-based study of nonattendance at screening for cardiovascular diseases and diabetes in older women: Nonattendees’ perspectives,” *J. Clin. Nurs.*, vol. 27, no. 5–6, pp. 939–948, 2018, doi: 10.1111/jocn.14018.

[72] D. J. T. Campbell, B. G. O’Neill, K. Gibson, and W. E. Thurston, “Primary healthcare needs and barriers to care among Calgary’s homeless populations,” *BMC Fam. Pract.*, vol. 16, no. 1, pp. 1–10, 2015, doi: 10.1186/s12875-015-0361-3.

[73] L. Sadler, R. Albrow, R. Shelton, H. Kitchener, and L. Brabin, “Development of a pre-notification leaflet to encourage uptake of cervical screening at first invitation: a qualitative study.,” *Health Educ. Res.*, vol. 28, no. 5, pp. 793–802, Oct. 2013, doi: 10.1093/her/cys103.

[74] J. Borrull-Guardeño, C. Sebastiá-Laguarda, F. Donat-Colomer, and V. Sánchez-Martínez, “Women’s knowledge and attitudes towards cervical cancer prevention: A qualitative study in the Spanish context,” *J. Clin. Nurs.*, vol. 30, no. 9–10, pp. 1383–1393, 2021, doi: 10.1111/jocn.15687.

[75] D. Llovet *et al.*, “Reasons For Lack of Follow-up Colonoscopy Among Persons With A Positive Fecal Occult Blood Test Result: A Qualitative Study,” *Am. J. Gastroenterol.*, vol. 113, no. August, pp. 1872–1880, 2018, doi: 10.1038/s41395-018-0381-4.

[76] S. Lou, M. Frumer, S. Olesen, A. H. Nielsen, and U. Væggemose, “Danish patients are positive towards fees for nonattendance in public hospitals. A qualitative study,” *Dan. Med. J.*, vol. 63, no. 7, pp. 7–10, 2016.

[77] M. Hussain-Gambles, R. D. Neal, O. Dempsey, D. A. Lawlor, and J. Hodgson, “Missed appointments in primary care: Questionnaire and focus group study of health professionals,” *Br. J. Gen. Pract.*, vol. 54, no. 499, pp. 108–113, 2004.

[78] J. Kue, L. A. Szalacha, M. B. Happ, and U. Menon, “Perceptions of cervical cancer and screening behavior among cambodian and Lao Women in the United States: An exploratory, mixed-methods study,” *J. Health Care Poor Underserved*, vol. 31, no. 2, pp. 889–908, 2020, doi: 10.1353/hpu.2020.0067.

[79] L. P. Wong, Y. L. Wong, W. Y. Low, E. M. Khoo, and R. Shuib, “Cervical cancer screening attitudes and beliefs of Malaysian women who have never had a pap smear: A qualitative study,” *Int. J. Behav. Med.*, vol. 15, no. 4, pp. 289–292, 2008, doi: 10.1080/10705500802365490.

[80] C. A. Smith-Miller, D. C. Berry, and C. T. Miller, “The Space Between: Transformative Learning and Type 2 Diabetes Self-Management,” *Hisp. Heal. Care Int.*, vol. 18, no. 2, pp. 85–97, 2020, doi: 10.1177/1540415319888435.

[81] S. Y. Oketch *et al.*, “Perspectives of women participating in a cervical cancer screening campaign with community-based HPV self-sampling in rural western Kenya: A qualitative study,” *BMC Womens. Health*, vol. 19, no. 1, pp. 1–10, 2019, doi: 10.1186/s12905-019-0778-2.

[82] F. I. Modibbo *et al.*, “Qualitative study of barriers to cervical cancer screening among Nigerian women,” *BMJ Open*, vol. 6, no. 1, 2016, doi: 10.1136/bmjopen-2015-008533.

[83] Y. Zhang, I. J. Ornelas, H. H. Do, M. Magarati, J. C. Jackson, and V. M. Taylor, “Provider Perspectives on Promoting Cervical Cancer Screening Among Refugee Women,” *J. Community Health*, vol. 42, no. 3, pp. 583–590, 2017, doi: 10.1007/s10900-016-0292-5.

[84] N. C. Raymond *et al.*, “Culturally informed views on cancer screening: A qualitative research study of the differences between older and younger Somali immigrant women,” *BMC Public Health*, vol. 14, no. 1, pp. 1–8, 2014, doi: 10.1186/1471-2458-14-1188.

[85] N. Rossell, J. Challinor, R. Gigengack, and R. Reis, “Choosing a miracle: Impoverishment, mistrust, and discordant views in abandonment of treatment of children with cancer in El Salvador,” *Psychooncology.*, vol. 26, no. 9, pp. 1324–1329, 2017, doi: 10.1002/pon.4302.

[86] C. Akre, P. A. Michaud, and J. C. Suris, “‘I’ll look it up on the Web first’: Barriers and overcoming barriers to consult for sexual dysfunction among young men,” *Swiss Med. Wkly.*, vol. 140, no. 23–24, pp. 348–353, 2010.

[87] O. Ogunsiji, L. Wilkes, K. Peters, and D. Jackson, “Knowledge, attitudes and usage of cancer screening among West African migrant women,” *J. Clin. Nurs.*, vol. 22, no. 7–8, pp. 1026–1033, 2013, doi: 10.1111/jocn.12063.

[88] P. Busingye, A. Nakimuli, E. Nabunya, and T. Mutyaba, “Acceptability of cervical cancer screening via visual inspection with acetic acid or Lugol’s iodine at Mulago Hospital, Uganda.,” *Int. J. Gynaecol. Obstet. Off. organ Int. Fed. Gynaecol. Obstet.*, vol. 119, no. 3, pp. 262–265, Dec. 2012, doi: 10.1016/j.ijgo.2012.06.015.

[89] C. A. Laranjeira, “Portuguese women’s knowledge and health beliefs about cervical cancer and its screening,” *Soc. Work Public Health*, vol. 28, no. 2, pp. 150–157, 2013, doi: 10.1080/19371918.2011.592042.

[90] L. P. Wong, Y. L. Wong, W. Y. Low, E. M. Khoo, and R. Shuib, “Knowledge and awareness of cervical cancer and screening among Malaysian women who have never had a Pap smear: a qualitative study.,” *Singapore Med. J.*, vol. 50, no. 1, pp. 49–53, Jan. 2009.

[91] T. E. Filade, E. O. Dareng, T. Olawande, T. A. Fagbohun, A. O. Adebayo, and C. A. Adebamowo, “Attitude to Human Papillomavirus Deoxyribonucleic Acid-Based Cervical Cancer Screening in Antenatal Care in Nigeria: A Qualitative Study,” *Front. Public Heal.*, vol. 5, no. September, pp. 1–10, 2017, doi: 10.3389/fpubh.2017.00226.
