# Supplementary material for: Understanding no-show behaviour for cervical cancer screening appointments among hard-to-reach women in Bogotá, Colombia: A mixed-methods approach
Source: PLoS One. 2022 Jul 22;17(7):e0271874. doi: 10.1371/journal.pone.0271874 (PMC9307170; doi:10.1371/journal.pone.0271874)
Supplement: Attachment — Submitted filename: Response to Reviewers.pdf [file pone.0271874.s003.pdf]


Editor

Comment:
Thank you for submitting your manuscript to PLOS ONE. After careful consideration, we feel that it has merit but does not fully meet PLOS ONE’s publication criteria as it currently stands. Therefore, we invite you to submit a revised version of the manuscript that addresses the points raised during the review process

Reply:
Thank you for your encouragement and the opportunity to review our work. We have made extensive revisions to the document which are detailed in this letter, which we are hopeful will satisfy the reviewers’ concerns.

Journal Requirements

When submitting your revision, we need you to address these additional requirements.

Comment 1:
Please ensure that your manuscript meets PLOS ONE's style requirements, including those for file naming.
Reply 1:
We have reviewed our manuscript to comply with format requirements.

Comment 2:
Thank you for including your ethics statement: 'SDS (2019EE47807), Pontificia Universidad Javeriana (FID-19-107) and the University of Southampton (ERGO ID 48583.A1) granted ethical approval for this study.'

(a) Please amend your current ethics statement to include the full name of the ethics committee/institutional review board(s) that approved your specific study.

(b) Once you have amended this/these statement(s) in the Methods section of the manuscript, please add the same text to the “Ethics Statement” field of the submission form (via “Edit Submission”).

For additional information about PLOS ONE ethical requirements for human subjects research, please refer to http://journals.plos.org/plosone/s/submission-guidelines#loc-human-subjects-research.

Reply 2:
We have included additional details on our methods section. Lines 92-95.

guidelines are followed. Pontificia Universidad Javeriana (Faculty of Engineering’s Research and Ethics Committee: FID-19-107), SDS (Ethics Committee for Health Research 2019EE47807) and the University of Southampton (Faculty of Social Sciences’ Ethics and Research Committee ERGO ID 48583.A1) granted ethical approval for this study.

Comment 3:
Please provide additional details regarding participant consent. In the ethics statement in the Methods and online submission information, please ensure that you have specified (1) whether consent was informed and (2) what type you obtained (for instance, written or verbal, and if verbal, how it was documented and witnessed). If your study included minors, state whether you obtained consent from parents or guardians. If the need for consent was waived by the ethics committee, please include this information.

Reply 3:
All participants granted informed consent during the phone call, and it was recorded. We have added a clarification on Section 2.3. Lines 174-176

> had previous training on working with vulnerable populations and discussing health-related topics. In each phone call, basic information of the project was provided, the patient was invited to take part of the study and oral informed consent was obtained. Patients authorized the conversations to be recorded. A research assistant
>
> 7

Comment 4:
In the ethics statement in the manuscript and in the online submission form, please provide additional information about the patient records used in your retrospective study, including: a) whether all data were fully anonymized before you accessed them; b) the date range (month and year) during which patients' medical records were accessed; c) the date range (month and year) during which patients whose medical records were selected for this study sought treatment; and d) the source of the medical records analyzed in this work (e.g. hospital, institution or medical center name).
Reply 4:
We have added additional details in Section 2.2. Lines 134-144

> We analysed data collected routinely by program managers (in SDS) to assess the performance of ACS. Between January 2017 and December 2019, appointments were scheduled for 23384 women aged between 21 and 65 years old. In each case, the outcome – show or no-show – was recorded. Table 1 presents the list of variables, grouped into two categories: patient and appointment-related information. We did not have access to poverty level data, marital status, or number of children for individual patients: these data are not held by SDS. For age and lead time we used decision trees to build categorical variables maximizing information value. This means that the categories (the number and the limits) were automatically selected by the algorithm to maximize inter-category difference and minimize intra-categories difference. This approach has also been found to generate more stable models [42]. SDS granted access to a fully anonymized database for our analysis. The data were accessed in August 2019 (all records from January 2017 to July 2019) and February 2020 (all records from August to December 2019). From this database, we randomly generated training (70%) and test (30%) sets.

Comment 5:
In your Methods section, please provide additional information about the participant recruitment method and the demographic details of your participants. Please ensure you have provided sufficient details to replicate the analyses such as: a) the recruitment date range (month and year), b) a table of relevant demographic details, c) a statement as to whether your sample can be considered representative of a larger population, d) a description of how participants were recruited, and e) descriptions of where participants were recruited and where the research took place.
Reply 5:
We have added additional details in Section 2.3. Lines 163-170

The aim of the qualitative phase is to understand the patient experience and reasons for health-seeking behaviour. Data were collected through semi-structured interviews using purposeful sampling [49,50]. We focused our analysis on patients with higher no-show risk, as their views can provide relevant information to design behavioural interventions [51]. Therefore, patients who met the following three eligibility criteria were considered: i) having received a home visit and an appointment scheduled between October and December of 2019 (3140 patients), ii) additionally, had been classified as a medium or high no-show risk according to the prediction models (1099 patients) and iii) additionally, had failed to keep their appointments (857 patients). Program managers provided a list of 100 randomly selected patients that met the criteria; we were able to reach 75 patients by phone and, of these, 15 declined to participate.

Five community workers collected data using phone interviews in Spanish, between January and February 2020. A nine-item interview guide was designed using relevant literature and discussed with public health

Comment 6:
To comply with PLOS ONE submission guidelines, in your Methods section, please provide additional information regarding your statistical analyses. For more information on PLOS ONE's expectations for statistical reporting, please see https://journals.plos.org/plosone/s/submission-guidelines.#loc-statistical-reporting.
Reply 6:
We have used RECORD (The REporting of studies Conducted using Observational Routinely-collected health Data) guidelines. We have also provided some additional information to clarify that our methods are reproducible and included the reference for the Python implementation. Lines 146-158.

To estimate the probability of attendance, two well-known machine learning models were implemented, Least Absolute Shrinkage and Selection Operator (LASSO) regression [43] and Random Forests (RF) [44]. Recent applications of LASSO in healthcare research include prediction of mortality rates [45] and medication adherence [46], among others. Additionally, for classification problems, RFs are less sensitive to outliers and eliminate the risk of overfitting [47] and thus improve the accuracy of the model. We conducted a parametric analysis on the penalization constant of the LASSO model and selected the one that maximizes the Area Under the Receiver Operating Curve (AUROC) while minimizing the number of selected variables. To validate the model, we randomly divided the training set into 10 groups, used nine groups for training, and the other for testing. Then, the testing group was iteratively changed, and the procedure was repeated ten times, resulting in 100 experiments. This is called a 10-by-10 cross validation process (10-by-10 CV). Next, we used the LASSO results to select the features included in the RF, optimized parameters using 30% of the training set and performed a 10-by-10 CV. The performance of both models was assessed using the average and standard deviation of the AUROC score over the 100 experiments. LASSO and RF Scikit-Learn's implementations were used for our analysis [48].

Comment 7:
We note that the grant information you provided in the 'Funding Information' and 'Financial Disclosure' sections do not match.
When you resubmit, please ensure that you provide the correct grant numbers for the awards you received for your study in the 'Funding Information' section.
Reply 7:
Thank you for the opportunity to clarify. The funding statement was included in the cover letter.

Comment 8:
Thank you for stating in your Funding Statement:
"The first author's research is partially funded by a PhD scholarship from the healthcare research stream of the program Colombia Científica – Pasaporte a la Ciencia, granted by the Colombian Institute for Educational Technical Studies Abroad (Instituto Colombiano de Crédito Educativo y Estudios Técnicos en el Exterior, ICETEX)."
Please provide an amended statement that declares *all* the funding or sources of support (whether external or internal to your organization) received during this study, as detailed online in our guide for authors at http://journals.plos.org/plosone/s/submit-now. Please also include the statement "There was no additional external funding received for this study." in your updated Funding Statement. Please include your amended Funding Statement within your cover letter. We will change the online submission form on your behalf.
Reply 8:
Thank you for the opportunity to clarify. The funding statement was included in the cover letter.

Comment 9:
Please upload a new copy of Figure 4 as the detail is not clear.
Reply 9:
Upon consideration, we have decided to eliminate Figure 4.

Comment 10:
We note that Figure 2 in your submission contain map images which may be copyrighted. All PLOS content is published under the Creative Commons Attribution License (CC BY 4.0), which means that the manuscript, images, and Supporting Information files will be freely available online, and any third party is permitted to access, download, copy, distribute, and use these materials in any way, even commercially, with proper attribution. For these reasons, we cannot publish previously copyrighted maps or satellite images created using proprietary data, such as Google software (Google Maps, Street View, and Earth). For more information, see our copyright guidelines: http://journals.plos.org/plosone/s/licenses-and-copyright.
Reply 10:
Upon consideration, we have decided to eliminate Figure 2.

Reviewer #1:

Comment 1:
Thank you for the opportunity review this article. It is important and its findings merit publication.
Reply 1:
Thank you for review and detailed feedback. We have addressed all your comments as explained below.

Comment 2:
This reviewer has a few concerns regarding the following broad areas:

(1) there are few details regarding the screening program eligibility criteria and these details are needed for interpretation of your results.
(2) the prediction model is interesting, however, given the limited variables used as inputs – the paper should de-emphasize the results of the model and underscore its use as way to identify women for the qualitative interviews.
(3) authors should focus more on the qualitative results and improve the interpretability of Figure 4 – currently, this reviewer does not understand its utility.
(4) the literature review is useful, however, please expand to include PubMed as I believe the authors are missing quite a few relevant pubs.

Reply 2:
Thank you for this summary. It was very helpful to understand the main concerns about our manuscript. We address each of these four points on the detailed comments below. Broadly, we have made the following changes:

(1) We included details of both the nation-wide screening program and the outreach program.
(2) We improved the description of the prediction algorithm.
(3) Upon consideration, we decided to eliminate Figure 4.
(4) We updated the review.

Comment 3:
The introduction includes the necessary points and rationale for conducting the study. However, it could be better organized. Please ensure that the final paragraph of the introduction outlines the objectives of the study. Lines 68-87 could be moved to the discussion.
Reply 3:
We agree that closing the Introduction with the aims of the study improves the transition to the Methods section. This paragraph is now on lines 80-86. However, to comply with journal's requirement of providing relevant literature within the Introduction, we have decided to keep lines 68-87 in this section.

> The aim of this study is to understand this no-show behaviour by combining prediction and interpretation approaches. The prediction approach is premised on the idea that it is possible to use routinely collected historical data to produce a numerical estimate of the no-show probability for each individual patient. However, the retrospective nature and limitations of such data make it impossible to identify the reasons that could lead to a missed appointment [24,25]. The aim of the interpretation approach is to understand the phenomenon by studying patients' perceptions and their decision-making processes [26]. Therefore, we use a qualitative approach to undertake an in-depth exploration of the perceived barriers to attendance [27].

Comment 4: Methods

Lines 110-111: Details regarding the program would be useful here such as: eligibility criteria for women, what areas of Colombia are included in this program (i.e. was it just Bogota or other cities in Colombia as well), how many women were eligible based on census data, what percentage of women were contacted for appointments, how were the woman contacted (door to door or via telephone, or both)? Regarding eligibility criteria for women – can the authors also comment on how they define low-income per the programs description?

Reply 4:

We have added a new Section 2.1. Lines 97-118

In Colombia, the cervical cancer screening program covers women between 25 and 65 years old, or younger in the presence of some risk factors [30]. Currently, this program primarily relies on Pap smear tests following a 1-1-3 scheme [31,32]. This means that women should undergo annual cytology tests, and then change to a three-year interval after two consecutive negative results. Additionally, the screening is included in the national health insurance scheme and hence no out-of-pocket payment is required. Nevertheless, different authors have reported quality problems with the cytology tests [33,34] and access inequalities for women living under conditions of poverty, in rural areas, or with low education levels [35,36]. In this context, recent legislation has adopted the Human Papilloma Virus (HPV) test for women between 30 and 65 years old, as screening strategy [31]. The gradual implementation of this new guideline should have started in 2019 and was expected to improve cancer control [37]. However, the National Ministry of Health assessed and identified operational and logistical barriers for the piloting phase and delayed its beginning to 2020 [38]. At the time of writing, we were not able to find any consolidated report about the HPV test piloting in the country.

In Bogotá, the cervical cancer screening component of ACS is designed to cover hard-to-reach women. For this program, SDS considers a woman to be hard-to-reach if despite being eligible, she has not undergone a Pap smear test over the last year. Additionally, to prioritize resource allocation for social programs, SDS uses a nation-wide adopted scoring system that classifies low-income citizens into four categories. The SISBEN[1] score ranges from 0 (extreme poverty) to 100 (wealthy) and is computed using self-reported information related to health, education, and housing, among others [39]. ACS covers approximately 18% of the population with the lowest SISBEN score [40]. On a monthly basis, the health insurance companies identify women who meet the criteria and enrol them in the home-visit based outreach program, managed by SDS. The number of home visits performed each month is highly variable. In 2019, for example, between 655 (January) and 1560 (May) women were visited monthly for community workers.

Comment 5: Methods

Line 110: of the 23,384 appointments scheduled, were these all unique patient appointments or might have there been repeats? If there are repeats, is it possible to get the number of unique appointments?

Reply 5:

We have added a clarification on Section 2.2. Lines 134-136

> We analysed data collected routinely by program managers (in SDS) to assess the performance of ACS. Between January 2017 and December 2019, appointments were scheduled for 23384 women aged between 21 and 65 years old. In each case, the outcome – show or no-show – was recorded. Table 1 presents the list of

Comment 6: Methods
Lines 128 – 131: Can you provide the number of women that fell into each category of the eligibility criteria so the reader can get a sense of each criterion?
Reply 6:
We have added details Section 2.3. Lines 163-168

> design behavioural interventions [51]. Therefore, patients who met the following three eligibility criteria were considered: i) having received a home visit and an appointment scheduled between October and December of 2019 (3140 patients), ii) additionally, had been classified as a medium or high no-show risk according to the prediction models (1099 patients) and iii) additionally, had failed to keep their appointments (857 patients). Program managers provided a list of 100 randomly selected patients that met the criteria; we were able to reach 75 patients by phone and, of these, 15 declined to participate.

Comment 7: Methods
Table 2: In Line 131, the text mentions 75 participants were included. However, Table 2 mentions 60 interviews. Please clarify this discrepancy.
Reply 7:
Although 75 were reached by phone, only 60 accepted our invitation to take part of the study. We have highlighted relevant information on section 2.2. Lines 167-169.

> Program managers provided a list of 100 randomly selected patients that met the criteria; we were able to reach 75 patients by phone and, of these, 15 declined to participate.

Comment 8: Methods
Table 3: Please provide a footnote that defines "Inductive category" clearly. From the text, I believe it means an emerging theme or an understudies topic.
Reply 8:
We agree this was not clear. We have added a footnote at the end of table 3. Line 222

> * A first order category is inductive if it emerged from the data and was not identified in the literature reviews.

Comment 9: Methods

Line 153: I am concerned that the SCOPUS database only retrieved 394 records when the authors searched cervical cancer screening and uptake. I did the same search in PubMed and found over 1500 results. Can the authors include PubMed given the comprehensive nature of this database? I find it difficult to believe that there were no papers that identified “cancer diagnosis” or “health” as a potential benefit of cancer screening.

Reply 9:

Many thanks for your comment and the opportunity to clarify. The main difference between the two results is that we searched using title and abstract of each paper. This change decreases the number of results from over 1500 to 535 using PubMed. Most of these 535 papers were either included in the SCOPUS data base or published after our submission. However, we updated the two reviews and made the following changes in the manuscript:

a. Added a clarification on Section 2.3. Lines 193-195.

> Two literature searches were conducted, using SCOPUS and PubMed databases, to identify deductive first-order categories. Fig 1 provides details of each review using the PRISMA guidelines [56]. We decided to limit our search using the title, abstract and key words option in SCOPUS and the title and abstract option in PubMed. First, we targeted journal papers, published in English, that use qualitative analysis in order to understand no-show

b. Updated Figure 1.

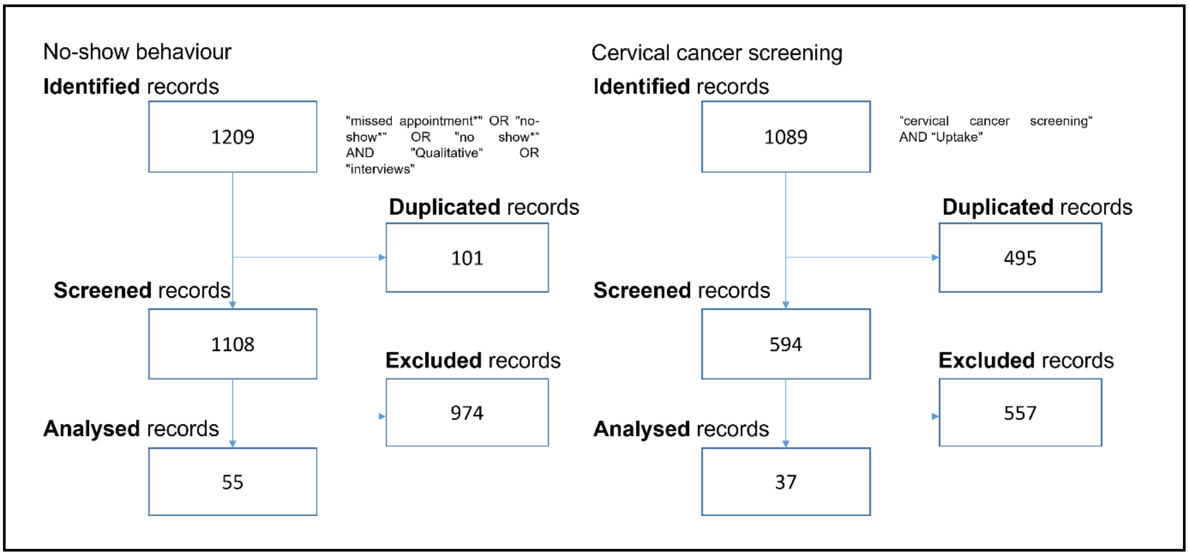

c. Updated the statistics on Section 2.3. Lines 196-199.

> behaviour in healthcare. We identified 55 papers published between 2004 and 2020. We note that of these 55, 40 were published after 2015 and only eight are on the topic of no-show behaviour in developing countries. Second, we aimed at identifying qualitative works studying cervical cancer screening uptake. We identified 37 papers published between 2005 and 2020. The majority of these works (62%) were conducted in developing country contexts.

Regarding the two inductive categories, many researchers have identified the lack of perceived benefits or the lack of knowledge as possible deterrents for screening uptake. This could be related to the "Health" or "Cancer diagnosis" categories. However, our participants talked specifically about these concepts during the interviews. This could be related to the fact that all these patients have already received the home visit to talk about the outreach program. Therefore, we decided to add independent first order categories in the framework.

Comment 10: Methods
Table 2: Can the authors describe what "conditions" means under protective behavior?
Reply 10:
We agree this particular term was unclear. We have changed first order category number 26 to "Non-compliance with requirements"

Second order	First order	
Barriers	Protective behaviour	
	25	Anxiety
	26	Non-compliance with requirements

Comment 11: Methods
Line 165 – typo
Reply 11:
We have made changes on line 207

207 and barriers. Therefore, we group the first-order categories using these constructs. Table 3 presents the resulting

Comment 12: Results
Lines 208-213: A comment here regarding the validity of the model is needed. The patient specific inputs are very few. More data regarding other factors such as marital status, number of children, employment status, educational level etc. are needed for this prediction model to be useful.
Reply 12:
Many thanks for your comment and the opportunity to clarify. We aimed at using routinely collected data to predict individual no-show probabilities. To ensure that this model can be used by SDS, it was important to limit the input variables to those that are available in their information system. However, the discrimination power and the stability of the results lead us to conclude that this model can be used to generate highly accurate predictions and support resource-allocation decisions.

In this context, we recognise that additional variables can be useful to improve the accuracy of the LASSO model. Additionally, we agree that our manuscript was lacking some details regarding the model to ensure that the reader can trust the results. Therefore, we have made the following changes:

a. We included a sentence recognizing the limitation to include other variables on Section 2.2. Lines 137-138.

137 variables, grouped into two categories: patient and appointment-related information. We did not have access to
138 poverty level data, marital status, or number of children for individual patients: these data are not held by SDS.

b. We improved the description of our quantitative methods on Section 2.2. Lines 146-158.

To estimate the probability of attendance, two well-known machine learning models were implemented, Least Absolute Shrinkage and Selection Operator (LASSO) regression [43] and Random Forests (RF) [44]. Recent applications of LASSO in healthcare research include prediction of mortality rates [45] and medication adherence [46], among others. Additionally, for classification problems, RFs are less sensitive to outliers and eliminate the risk of overfitting [47] and thus improve the accuracy of the model. We conducted a parametric analysis on the penalization constant of the LASSO model and selected the one that maximizes the Area Under the Receiver Operating Curve (AUROC) while minimizing the number of selected variables. To validate the model, we randomly divided the training set into 10 groups, used nine groups for training, and the other for testing. Then, the testing group was iteratively changed, and the procedure was repeated ten times, resulting in 100 experiments. This is called a 10-by-10 cross validation process (10-by-10 CV). Next, we used the LASSO results to select the features included in the RF, optimized parameters using 30% of the training set and performed a 10-by-10 CV. The performance of both models was assessed using the average and standard deviation of the AUROC score over the 100 experiments. LASSO and RF Scikit-Learn's implementations were used for our analysis [48].

c. We improved our results including comments on the performance of the models. Section 3.1, lines 229-239

Table 4 presents the results of the LASSO regression model. For classification proposes, a value of one is assigned to those patients attending their appointments. Therefore, higher odds ratios mean lower no-show probabilities. For each variable, we report the average, the 5th percentile and the 95th percentile of the regression coefficient and the average Odds ratio, obtained through 100 experiments. This model has a moderate discriminatory power, and its results are not sensitive to the sample. The average AUROC score is 0.65 with a standard deviation of 0.001. This could indicate that the non-linear component of the relationship between the variables and the no-show probability is high. It is also possible that including additional patient information could lead to better performance. Variables such as income and education levels have been found to be good predictors of attendance for cervical cancer screening [144,145]. However, our aim was to leverage routinely available data to inform patient prioritization by SDS. Therefore, the LASSO results are used to understand the characteristics of patients with higher no-show risk and to select the variables that should be used in the RF model.

d. We included information about the variability of the coefficients on Table 4

Table 4: Results of the LASSO regression model.

Variable	Coefficient			Odds Ratio
	Average	Percentile 5th	Percentile 95th	Average
Age (years)				
[21, 27]	-0.82	-0.85	-0.79	**0.44**

Comment 13: Results
Table 4: Is it possible to create more categories of Poverty? Why did the authors decide to create only a binary variable? It would be important to show that high poverty areas were less likely to attend.
Reply 13:
We used decision trees to automatically build the categories (the number and the limits) for each variable. We have added details on Section 2.2. Lines 139-142.

For age and lead time we used decision trees to build categorical variables maximizing information value. This means that the categories (the number and the limits) were automatically selected by the algorithm to maximize inter-category difference and minimize intra-categories difference. This approach has also been found to generate more stable models [42]. SDS granted access to a fully anonymized database for our analysis. The data were

Comment 14: Results
Line 215: What is "RF"?
Reply 14:
We have ensured that the abbreviation was defined the first time we used it in section 2.3 (line 147) and included the full name in section 3.1 (line 261).

Fig 2 shows the outcome when patients are assigned, in decreasing order of no-show probability (calculated in three different ways: by LASSO, by Random Forest, and at random) to different sizes of intervention

Comment 15: Results
Figure 3 is not useful. I am not sure how to interpret it given how unreliable the model is. I would suggest the authors remove this and delete text 214 – 223.
Reply 15:
We hope that the changes we have made to improve the description of our modelling approach address the question of the reliability of our results. We have also made changes on Figure 3 (now Figure 2).

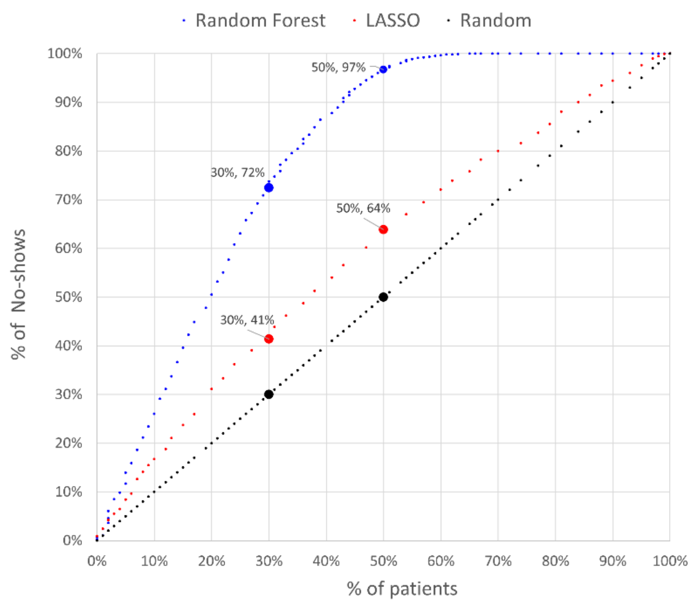

Comment 16: Results

Figure 4 is very blurry. Can the authors provide some footnotes on Figure 4 to explain why it is arranged the way it is? Do the boxes mean something? Is there a reason some boxes are shaped differently than others? The figure is not self explanatory and should be able to stand alone.

Reply 16:

Upon consideration we decided to eliminate this figure. We have added a column in Table 5 presenting the frequency of each category in our data.

Table 5: Quotes from the interviews

N	Category	Quote	Frequency
2	Category: Barriers - Access<br>Inconvenient appointment slots	*I would say that [it is important to have] more service time. Sometimes you go to work at five or four thirty in the morning and you are back home at seven p.m. There is not service at nights and weekend appointments are always booked. It is difficult to keep an appointment*	5

Comment 17: Discussion

Line 271; Summary of overall main findings would be useful here.

Reply 17:

We have added a new Section 4.1. Lines 320-335

4.1. Main findings

Using routinely collected data, we were able to accurately predict individual no-show probabilities for cervical cancer screening appointments in Bogotá. First, we fitted a LASSO regression model to identify the characteristics of the higher no-show risk appointments. We found that younger patients living in zones with higher poverty levels are less likely to attend. Additionally, offering short lead times and Sunday appointments could increase screening uptake among hard-to-reach women in the city. Next, we used the LASSO results to select the variables to train a RF aimed at improving prediction accuracy. The resulting model has a good discrimination power and low variability in its performance (Average AUROC score 0.84 and standard deviation of 0.01). We used the RF results to inform the sample selection for a series of semi-structured interviews.

We interviewed 60 hard-to-reach women who received a home visit from the outreach program and had failed to attend their cytology appointments. Although most patients perceived the home visits to be informative, they found it hard to navigate the service delivery process and experienced access barriers. Qualitative data also enhanced the interpretation of the quantitative results. For example, the LASSO results show a relationship between the appointment date and the no-show probability. In the same vein, during the interviews some patients expressed that taking time off from work or childcare responsibilities might act as deterrents for screening uptake.

Comment 18: Discussion
Line 329: Define SDS
Reply 18:
We have ensured that the abbreviation was defined the first time we used it in section 1 (line 53) and included the full name in section 4.3 (line 391).

> An impact evaluation should support future decision-making. After three years of running the outreach program, the District Secretariat of Health (*Secretaría Distrital de Salud*, SDS) has sufficient information to quantify

----
Reviewer #2:

Comment 1:
Thank you for the opportunity to review this manuscript. This is a well written manuscript with an interesting research question and methodological approach. It is great to see mixed-methods approach being used in this setting. The authors have done a nice job in describing the methods and results. Non-compliance to cervical cancer screening is furthermore an important topic to address and understand, in order to design future policies to improve compliance rates.
Reply 1:
Thank you for your encouragement and positive feedback. We have addressed all your comments in detail as explained below.

Comment 2:
For the qualitative interviews, what questions were asked? How did you design the interview guide/questions?
Reply 2:
We have added details in Section 2.4. Lines 169-171

> Five community workers collected data using phone interviews in Spanish, between January and February 2020. A nine-item interview guide was designed using relevant literature and discussed with public health specialists and community workers at SDS, in one workshop (see S1 Appendix). Before starting data collection,

Comment 3:
In Table 3, could you add more description to the first order categories? These are interesting and may be useful to others; however, while some are self explanatory, others are not.
Reply 3:
We agree this would help, and have decided to include a new Appendix with the definition of all first order categories. We refer to the appendix on line 208.

> and barriers. Therefore, we group the first-order categories using these constructs. Table 3 presents the resulting 44 categories of the analytical framework. A description of each category is provided in Appendix S2. The ten

Comment 4:
Results Table 4, could you elaborate what drives the differences between days and months?
Reply 4:
We discussed this in Sections 3.1 (Lines 246-250) and 4.2 (Lines)

Table 4 also shows a relationship between appointment-related variables and no-show probability. Regarding the appointment month, school vacation periods (January, March, June, and December) have lower odds ratios. Additionally, while patients are more likely to attend appointments on Sundays, Saturday appointments have a higher no-show risk. This might indicate that the requirement to take time off from work could act as a barrier to cytology uptake. Lastly, we find that longer lead times increase the risk of no-show.

primary care appointments. The existence of patterns in no-show probabilities according to the month of the year or day of the week has been documented previously [155–158]. Two qualitative results might offer a context for these quantitative findings. First, a lower no-show risk on Sundays might be explained by the difficulties reported by some participants in taking time off work. Second, some participants stated that their childcare responsibilities caused them to miss appointments, which could explain why the no-show risk was higher during the school vacation months.

Comment 5:
The discussion section would benefit from a brief summary of main findings at the beginning, including a discussion of how the qualitative and quantitative methods complemented each other.
Reply 5:
We have added a new Section 4.1. Lines 320-333.

4.1. Main findings

Using routinely collected data, we were able to accurately predict individual no-show probabilities for cervical cancer screening appointments in Bogotá. First, we fitted a LASSO regression model to identify the characteristics of the higher no-show risk appointments. We found that younger patients living in zones with higher poverty levels are less likely to attend. Additionally, offering short lead times and Sunday appointments could increase screening uptake among hard-to-reach women in the city. Next, we used the LASSO results to select the variables to train a RF aimed at improving prediction accuracy. The resulting model has a good discrimination power and low variability in its performance (Average AUROC score 0.84 and standard deviation of 0.01). We used the RF results to inform the sample selection for a series of semi-structured interviews.

We interviewed 60 hard-to-reach women who received a home visit from the outreach program and had failed to attend their cytology appointments. Although most patients perceived the home visits to be informative, they found it hard to navigate the service delivery process and experienced access barriers. Qualitative data also enhanced the interpretation of the quantitative results. For example, the LASSO results show a relationship between the appointment date and the no-show probability. In the same vein, during the interviews some patients expressed that taking time off from work or childcare responsibilities might act as deterrents for screening uptake.

Comment 6:
In the discussion of implications and future policies, could HPV self-sampling be an option? Home-based self-collection of samples is currently being rolled out worldwide as an approach to reach underscreened women. Is this relevant for this setting? What is the status of HPV testing?
Reply 6:
Many thanks for your observation. We have made the following two changes.

a) We added a paragraph on Section 2.1, describing the status of the screening program including new legislation on HPV testing. Lines 97-108.

2.1 Study context

In Colombia, the cervical cancer screening program covers women between 25 and 65 years old, or younger in the presence of some risk factors [30]. Currently, this program primarily relies on Pap smear tests following a 1-1-3 scheme [31,32]. This means that women should undergo annual cytology tests, and then change to a three-year interval after two consecutive negative results. Additionally, the screening is included in the national health insurance scheme and hence no out-of-pocket payment is required. Nevertheless, different authors have reported quality problems with the cytology tests [33,34] and access inequalities for women living under conditions of poverty, in rural areas, or with low education levels [35,36]. In this context, recent legislation has adopted the Human Papilloma Virus (HPV) test for women between 30 and 65 years old, as screening strategy [31]. The gradual implementation of this new guideline should have started in 2019 and was expected to improve cancer control [37]. However, the National Ministry of Health assessed and identified operational and logistical barriers for the piloting phase and delayed its beginning to 2020 [38]. At the time of writing, we were not able to find any consolidated report about the HPV test piloting in the country.

b) We made changes on Section 4.2, including a recent review on self-sampling for HPV testing. Lines 398-400

> health outcomes in low resourced health systems [177]. For example, a recent review concluded that self-sampling approaches have been found to increase acceptability of cervical cancer screening [178]. In this context, by
