# Supplementary material for: Understanding no-show behaviour for cervical cancer screening appointments among hard-to-reach women in Bogotá, Colombia: A mixed-methods approach
Source: PLoS One. 2022 Jul 22;17(7):e0271874. doi: 10.1371/journal.pone.0271874 (PMC9307170; doi:10.1371/journal.pone.0271874)
Supplement: Attachment — Submitted filename: Response to the reviewers.pdf [file pone.0271874.s004.pdf]


Editor

Comment:
Thank you for submitting your manuscript to PLOS ONE. After careful consideration, we feel that it has merit but does not fully meet PLOS ONE's publication criteria as it currently stands. Therefore, we invite you to submit a revised version of the manuscript that addresses the points raised during the review process

Reply:
Thank you for your encouragement and the opportunity to review our work. We have made revisions to the document which are detailed in this letter. We are hopeful this version of the manuscript will satisfy the reviewers' concerns.

Reviewer #3

Comment 1:
Thank you for the opportunity to review the revised version of this manuscript. The authors' responses to the comments from previous reviewers were satisfactory. This exploratory sequential mixed methods study was meticulously conducted. The quantitative component interestingly applied machine learning techniques, the qualitative component was properly conducted, whereas the systematic review was adequately performed using two major literature databases, all of which were presented in compliance with standard reporting guidelines.

Reply 1:
Thank you for your assessment and positive feedback. We are glad to know that you found our responses satisfactory. We've made changes to address your concern.

Comment 2:
I strongly believe that the references could be more concise—at least by moving several of them in the systematic review component to be supplementary file.

Reply 2:
Upon consideration, we agree that it is possible to place the references supporting the first-order categories in a supplementary file, without making sacrifices on the overall quality of the paper. Hence, we have made two changes:

1. We changed Table 3 to present only the Conceptual Framework. Page 10.

Table 3: Analytical Framework Categories

Second order	First order
Barriers	Access
	1 Financial stress
	2 Inconvenient appointment slots
	3 Long lead times
	4 Geographical access
	5 Work Commitments

2. We have added Table 2 to the Appendix S2

44 categories of the analytical framework. Additionally, a description of each category and the list of references supporting the deductive categories are provided in Appendix S2. The ten inductive categories were included at

Table2: References supporting the first order categories

Second order	First order	No-Show behaviour studies	Cervical cancer screening studies
Barriers	Access		
	1 Financial stress	[1], [2], [11], [12], [3]–[10]	[13], [14], [23], [15]–[22]

Reviewer #4

Comment 1:
The authors have done an excellent job of responding to reviewer comments and the revisions have resulted in a presentation of the results that is clearer to the reader. There are a few minor points that I think would improve the structure and flow of the paper.

Reply 1:
Thank you for assessment and the opportunity to review our work. In what follows, we address your concerns.

Comment 2:
Lines 97-118 have been added in response to Reviewer 1; these are quite lengthy and can be summarized to focus on the clinical guidelines and current situation that may impact the study question and outcomes.

Reply 2:
Upon consideration we agree that some of the details could be deleted. We have rewritten this section to convey two main messages: i) the way the screening program works in Colombia and ii) the definition of hard-to-reach women adopted in Bogotá.

2.1 Study context

In Colombia, the cervical cancer screening program covers women between 25 and 65 years old, or younger in the presence of some risk factors [30]. Currently, this program primarily relies on Pap smear tests following a 1-1-3 scheme [31,32]. This means that women should undergo annual cytology tests, and then change to a three-year interval after two consecutive negative results. Additionally, the screening is included in the national health insurance scheme and hence no out-of-pocket payment is required. Recent legislation has adopted the Human Papilloma Virus (HPV) test for women between 30 and 65 years old, as screening strategy [31]. However, at the time of writing, we were not able to find any consolidated report about the HPV test piloting in the country.

In Bogotá, the cervical cancer screening component of ACS is designed to cover hard-to-reach women. For this program, SDS considers a woman to be hard-to-reach if despite being eligible, she has not undergone a Pap smear test over the last year. Additionally, to prioritize resource allocation for social programs, SDS uses a nation-wide adopted scoring system that classifies low-income citizens into four categories. The SISBEN[1] score ranges from 0 (extreme poverty) to 100 (wealthy) and is computed using self-reported information related to health, education, and housing, among others [33]. ACS covers approximately 18% of the population with the lowest SISBEN score [34].

Comment 3:

The results section (Starting in line 229) would be stronger if the authors started with a more clear description of their findings, rather than an explanation of the model. Lines 229-232 can all be placed in the methods.

Reply 3:
We have placed lines 229-232 in the methods section.

Operating Curve (AUROC) while minimizing the number of selected variables. For classification proposes, a value of one was assigned to those patients attending their appointments. Therefore, higher odds ratios mean higher attendance probabilities.

6

Table 4 presents the results of the LASSO regression model. This model has a moderate discriminatory power, and its results are not sensitive to the sample. The average AUROC score is 0.65 with a standard deviation of 0.001. This could indicate that the non-linear component of the relationship between the variables and the no-

Comment 4:
Throughout the methods, there are opportunities to more clearly present what the findings show. Have the authors considered rephrasing the findings so that the odds ratios represent a greater odds of attending appointments? Rather than the counterintuitive "higher odds ratio=lower no show." The other option is to flip the outcome variable, so that no-show is the outcome of interest, and the odds ratios would be flipped (i.e. a greater odds ratio would mean a greater odds of no-show).

Reply 4:
You are right. To avoid any confusion, as the attendance was coded with 1, we have made changes through the paper stating that we aim at predicting attendance probabilities. This way, the aim and the variable representation are coherent.
